# *KAT6B* Genetic Variant Identified in a Short Stature Chinese Infant: A Report of Physical Growth in Clinical Spectrum of *KAT6B*-Related Disorders

**DOI:** 10.3389/fped.2020.00124

**Published:** 2020-04-23

**Authors:** Liuyan Zhu, Lina Lv, Dingwen Wu, Jie Shao

**Affiliations:** ^1^Department of Pediatric Health Care, The Children's Hospital, Zhejiang University School of Medicine, Hangzhou, China; ^2^National Clinical Research Center for Child Health, Hangzhou, China; ^3^Department of Genetics and Metabolism, The Children's Hospital, Zhejiang University School of Medicine, Hangzhou, China

**Keywords:** *KAT6B*, SBBYSS, GTPTS, short stature, *de novo* variant

## Abstract

Say-Barber-Biesecker-Young-Simpson syndrome (SBBYSS, OMIM#603736) and genitopatellar syndrome (GTPTS, OMIM#606170), characterized by global developmental delay/intellectual disability and special clinical manifestations, are two distinct clinically overlapping syndromes caused by truncating sequence variants in the *KAT6B* (10q22.2) gene. We detected a *de novo* heterozygous variant within exon 16 of *KAT6B* (Chr10p: 76781966-76781967) in a 7-months-old female infant who showed symptoms of short stature, global developmental delay, blepharophimosis, and lacrimal duct anomalies highly consistent with SBBYSS. Following the clinical features, we analyzed the *KAT6B* gene using Next Generation Sequencing (NGS) techniques. Her parents didn't present the same genetic variant. The patient we reported here is mainly characterized by syndromic forms of short stature and developmental delay, which may contribute to the understanding of clinical genetics for *KAT6B*-associated disorders.

## Background

*KAT6B*-related disorders, including both Say-Barber-Biesecker-Young-Simpson syndrome (SBBYSS, OMIM#603736) and genitopatellar syndrome (GTPTS, OMIM#606170), are caused by distinct variants in the KAT6B (OMIM#605880) gene. All variants are *de novo* dominant mutations resulting from nonsense or frame-shifts that lead to protein truncation. Both phenotypes are characterized by significant global developmental delay/intellectual disability and congenital anomalies, such as hypotonia, genital abnormalities in males (cryptorchidism), and patellar hypoplasia/agenesis (1). The degree of global developmental delay/intellectual disability is variable, from mild to moderate. Some patients have speech delay despite normal hearing (2). In addition, congenital heart defects, dental abnormalities, hearing loss, and thyroid anomalies are common to both phenotypes. In SBBYSS, blepharophimosis/ptosis, lacrimal duct anomalies, a bulbous nasal tip and mask-like face, extremity joint laxity, long thumbs/big toes are often observed (3).

However, GTPTS usually shows more severe clinical features, including agenesis of the corpus callosum with microcephaly, flexion contractures of the hips and knees, absent patellae, hydronephrosis, and/or multiple renal cysts (4, 5).The common phenotypes and unique characteristics of GTPTS and SBBYSS are presented in Table 1 (3). Feeding difficulties are more likely to be early signs at birth. The diagnosis is mainly dependent on clinical features (phenotype) and genetic tests (genotype).

**Table 1:** The common phenotypes and unique characteristics of GTPTS and SBBYSS.

**Classification**		**GTPTS**	**SBBYSS**
Common phenotypes	Neurological features	Global developmental delay/intellectual disability	
	Facial features	Blepharophimosis / ptosis, prominent cheeks, nose with either a bulbous tip or a broad or prominent base	
	Skeletal malformations	Patellar anomalies	
	Genital, cardiac anomalies	Cryptorchidism; atrial septal defects, ventricular septal defects and a patent foramen ovale	
	Others	Feeding difficulties, thyroid anomalies, dental anomalies, hearing loss	
Unique characteristics	Neurological features	Microcephaly, thin or absent corpus callosum	Smaller than average head circumference
	Facial features	Some degree of blepharophimosis and ptosis, others are not considered so distinctive as in SBBYSS	Mask-like face, lacrimal duct abnormalities, cleft palate
	Respiratory features	Respiratory difficulties	Rare
	Skeletal features	Flexion contractures at hips and knees, radiological anomalies in pelvis, spine and ribs, club feet	Long thumbs and great toes
	Anal and genital anomalies	Anal atresia or stenosis, rectal duplication, anteriorly positioned anus, clitoromegalymand/or hypoplasia of the labia, scrotal hypoplasia	Rare
	Renal anomalies	Hydronephrosis, multiple renal cysts	Vesicoureteric reflux

Short stature or failure to thrive in childhood is a frequent reason for referral to pediatric sub-specialists (6). Growth is a complex process influenced by genetic, hormonal, nutritional, environmental factors, or chronic systemic diseases, both pre- and postnatally (7). Various genetic syndromes, including Turner syndrome, Prader-Willi syndrome or Noonan syndrome, often present with growth defects and short stature (8). Thus, a genetic test may be an appropriate way (9) for differential diagnoses of those children with a pathological growth impairment. Yet, few reports presented short stature or failure to thrive as signs of *KAT6B*-related disorders. Here, we report a Chinese case of *KAT6B*-related disorders. The patient whom we followed up was characterized by a short stature associated with special clinical symptoms.

## Materials and Methods

### Case Presentation

A 7-months-old girl was referred to the Department of Pediatric Health Care, Children's Hospital Zhejiang University School of Medicine in January 2018 because of failure to thrive. The patient was born from non-consanguineous Chinese parents with no family history of congenital anomalies. No abnormalities were identified by prenatal ultrasonography and newborn screening. She was the first living child (G2P1). The first fetus (G1P0) was spontaneously aborted at the gestational age of 3 months. The girl was born at 40 weeks with 2,850 g (z-score: −0.7) of birth weight, 48 cm (z-score: −0.6) of birth length and 31.5 cm (z-score: −2.0) of head circumference. After birth, she presented weak crying and feeding difficulties. Her mother and father were respectively 26 and 27 years old when she was born. Their heights were 150 and 175 cm respectively.

The girl was breast fed. She occasionally had some overflow but with no history of choking cough or frequent vomiting. She showed slower physical growth velocity than the other girls at the same age.

Anthropometric measures presented as follows: body weight was 5.2 kg (z-score: −2.5), length was 60 cm (z-score: −3.1), and head circumstance was 41.2 cm (z-score: −1.2), assessed according to the 2006 WHO international growth standard charts. Revised Gesell Developmental Schedules were used to evaluate her developmental quotient (DQ), which showed developmental delays with average DQ as low as 52 scores.

**Figure 1:**
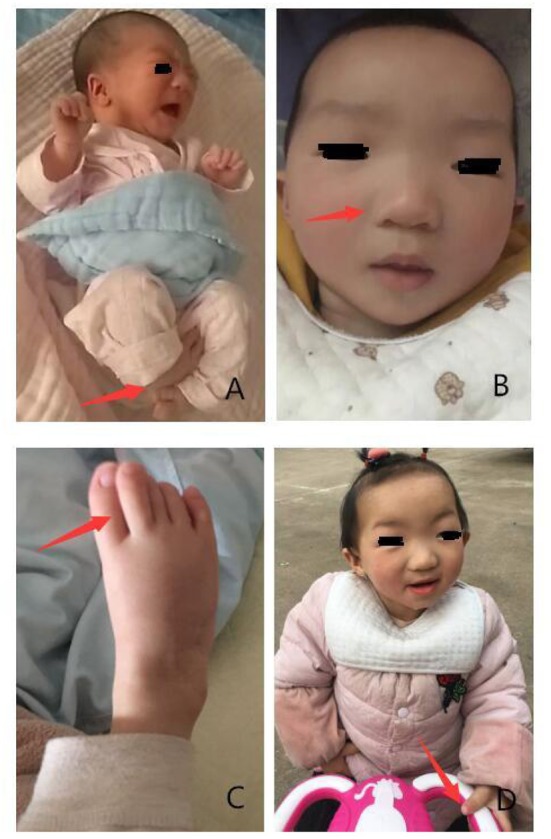
Phenotypes presented in the patient with *KAT6B* genetic variant. **(A)** Congenital horseshoe varus foot at birth. **(B,C)** Narrow palpebral fissure, ptosis, a broad nasal bridge, bulbous nasal tip, small mouth, thin upper lip, great toes. **(D)** Stand up with assistance when she was 19 months.

Following physical examination, we found that she had skeletal anomalies and joint limitations with congenital horseshoe varus foot. Moreover, facial deformity was manifested as narrow palpebral fissures, ptosis or flagging eyelids, a broad nasal bridge with a bulbous nasal tip, a small mouth, thin upper lip, and small, low set ears (5, 10) (Figure 1). There were no anomalies with her anal and genitals. Muscle tone of limbs showed a little higher.

Cardiac ultrasonography confirmed atrial septal defect (0.36 cm diameter) with mild tricuspid valve regurgitation. Abdominal ultrasonography demonstrated the size of the left kidney as 4.6^*^2.5 cm, and the right kidney as 5.0^*^2.2 cm. Both of them were relatively small. On the contrary, the pancreas was full (a little bigger than usual). Pelvic X-ray showed big tip angles of the acetabulum slants, with partial shallow of the acetabulum form (left 27°, right 25°). The bilateral knee joints in the orthotopic film of X-ray, brain magnetic resonance imaging (MRI) and electroencephalogram (EEG) showed no obvious abnormalities. Her auditory brainstem response (ABR) showed 25dBnHL in the left and 20dBnHL in the right ear. No obvious abnormal signs were found in her long bone of the limbs in the X-ray film. Blood tests including renal function, liver function, electrolytes, and thyroid function had no positive findings. Standard karyotyping was 46,XX. Considering her growth failure, global developmental delay and clinical features together, we obtained informed consent from the parents for genetic testing. The study was approved by the ethics committee of the Children's Hospital, Zhejiang University School of Medicine (2019-IRB-157).

### Targeted Next Generation Sequencing and Data Analysis

The genomic DNA of the patient and her parents was isolated from 2-ml peripheral blood samples using QIAamp DNA Blood Mini Kit (Qiagen GmbH, Hilden, Germany). Qubit dsDNA detection kit and Qubit4 fluorometer (Invitrogen, Carlsbad, CA, USA) were used to detect DNA concentration and purity. An adapter-ligated library was produced with Agilent Sure Select Target Enrichment System (Agilent Technologies Inc., Santa Clara, CA, US) according to the manufacturer's instructions. The capture library was performed using an XT Inherited Disease Panel (cat No. 5190–7519, Agilent Technologies Inc.) containing 2,742 genes. Clusters were then generated by isothermal bridge amplification using an Illumina cBot Station, and sequencing was performed on an Illumina HiSeq 2500 System (Illumina Inc., San Diego, CA, US). Using human genome hg19 as the reference, alignment of sequence and repeated labeling were performed using BWM version 0.7.17 (http://bio-bwa.sourceforge.net/) and Picard bioinformatics software version 2.5.0 (https://broadinstitute.github.io/picard/) for biological analysis and interpretation. GATK 4.0.0.0 (https://gatk.broadinstitute.org/hc/en-us/sections/360007407851-4-0-0-0) and Samtools 1.8 (https://sourceforge.net/projects/samtools/files/samtools/1.8/) were used to identify mutation sites.

### Sanger Sequencing Verification of the *KAT6B* Gene

The variants in the *KAT6B* gene were detected using Sanger sequencing for the patient and her parents. PCR was performed according to TaKaRa LA PCR™ Kit ver.2.1 (TaKaRa, Dalian, China), with special primers for the amplification of *KAT6B* gene. The PCR products (5 μL) were examined and purified using BigDye® Terminator v3.1 Cycle Sequencing Kit (Invitrogen, Paisley, UK). The resulting DNA was sequenced on ABI 3500XL platform (Applied Biosystems; Thermo Fisher Scientific Inc., Waltham, MA, US). The sequence data were analyzed with the SeqMan Pro software version11.1.0 (DNASTAR).

## Results

### Genetic Testing

**Figure 2:**
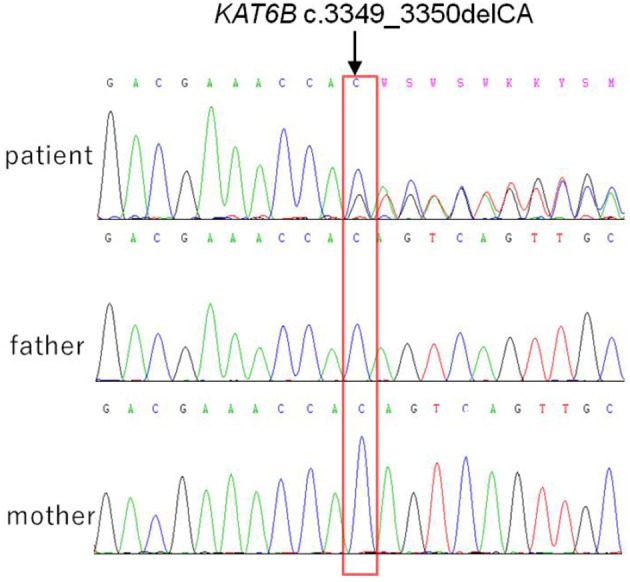
*De novo KAT6B* genetic variant identified in the patient, which is c.3349_3350delCA in exon16, her parents were normal.

The *KAT6B* gene sequencing revealed the heterozygous frame-shift variant c.3349_3350delCA (p.Q1117Vfs^*^19) in exon 16 (Figure 2). This variant was found in neither the ExAC nor GnomAD, 1000G databases. According to the standards and guidelines for the interpretation of sequence variants issued by the American College of Medical Genetics (ACMG) (11), this frame-shift mutation is pathogenic. The sequencing results demonstrated that the parents were normal (Figure 2), indicating that the variant in the patient was *de novo*.

### Follow-Up of Growth and Development

**Figure 3:**
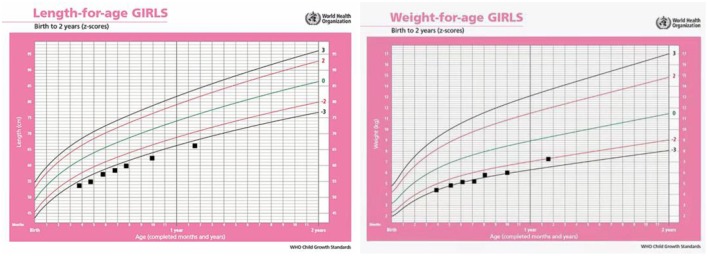
Growth chart of the patient from 4 to 19 months.

The girl presents severe delays in physical growth and mental development. Teeth began to erupt at 9 months of age. Rolling over appeared at 11 months and crawling at 15 months. At 19 months old, she had 16 teeth. Her weight, length and head measurements were 7.8 kg (z-score: −1.9), 69 cm (z-score: −4.2), and 44 cm (z-score: −1.7), respectively. She was just able to stand up with assistance and made a few steps in support (Figure 1D). Her language was limited to repetitive use of single vowels and consonant syllables. The growth curve of the patient is shown as below (Figure 3).

## Discussion

SBBYSS or GTPTS syndrome is a rare condition due to the *KAT6B* genetic variant. The *KAT6B* gene is located on chromosome 10q22.2, containing 20 exon and encoding lysine acetyltransferase 6B, as a part of the histone H3 acetyltransferase complex. *KAT6B*-related disorders are inherited in an autosomal dominant manner (12, 13). The prevalence of SBBYSS and GTPTS is still not accurately known.

According to the analysis of genotype-phenotype correlations, the clinical phenotypes of SBBYSS or GTPTS vary depending on the locations of the variants in *KAT6B* regions (5). The mutations of GTPTS are more likely to be located in the proximal portion (5′) of the last exon 18 (nucleotide positions c.3680_4368), and lead to the expression of a protein without a C-terminal portion (5, 6). In contrast, SBBYSS mutations tend to locate in exon 15–18, mostly in the distal portion (30′) of exon 18 [the last exon (nucleotide positions c.4069_5734)] or exceptionally in earlier exons, leading to nonsense-mediated decay and no protein production (6). Thus, the clinical features overlapping both GTPTS and SBBYSS are likely to result in either haploinsufficiency or the loss of a function normally mediated by the C-terminal region of the acidic domain (3). However, they have their unique features. The variant found in our patient was located at 10q22.2, nucleotide positions c.3349_3350 (p.Q1117Vfs^*^19), which supports the causal relationship between clinical aspects we found and the genetic variant.

To the best of our knowledge, this is the first Chinese case that implicates short stature with mental delay and the typical phenotypes of SBBYSS or GTPTS, since there was only one study that had reported short stature as being associated with the *KAT6B* genetic variant, but without mental retardation or clinical features (14). In the previous reported case, a Chinese boy, who presented with short stature, delayed bone age, and growth hormone deficiency, was screened using targeted next generation sequencing, and a *de novo* novel nonsense pathogenic mutation in exon14 of the *KAT6B* gene at position c.2636T>A(p.Leu879X) was found. It implied that the variants in *KAT6B* gene may present short statue or growth failure in phenotype.

As a component of the histone H3 acethytransferase complex, *KAT6B* was found to be highly expressed in adult neural stem cells and strongly expressed in the diaphysis of the long bones and the patella (13, 15). In mice, defects in the *KAT6B* gene lead to skeletal and brain developmental defects, and failure to thrive (16). Few reports revealed whether variants in the *KAT6B* gene have any correlation with growth hormone deficiency (GHD) or pituitary dysfunction. Growth hormone (GH)-insulin-like growth factor (IGF)-I axis is a key procedure in the endocrine mechanism which regulates linear growth in children. Previous studies showed that *KAT6B* regulated the Ras-mitogen-activated protein kinase (RAS-MAPK) signaling pathway through H3 acetylation (17). Then, the variants in the *KAT6B* gene may result in hyperphosphorylation of proteins in the RAS-MAPK signaling pathway. In a mouse model of Noonan syndrome, it has been proved that activating mutations of the tyrosine phosphate SHP2 inhibits GH-induced IGF-1 release through RAS-MAPK/ERK1/2 hyperactivation, which could contribute to growth retardation (18). Interestingly, a patient with a Noonan syndrome-like phenotype, who mainly presented with a feature of short stature, blepharoptosis, was found to have haploinsufficiency of the *KAT6B* gene, which resulted in 50% reduction in *KAT6B* expression in the patient (17).

In our case, the variant (c.3349_3350delCA) was identified in exon 16 in the *KAT6B* gene, which was not occurred in her parents. The clinical phenotype presented with major features of SBBYSS and several minor GTPTS characteristics, as well as a significant growth failure. We consider it as a mixed phenotype but with more tendency to SBBYSS. It implicated that the phenotypes associated with *KAT6B* disease causing variants may present with growth failure or short stature, together with the common features of *KAT6B*-related disorders. In practice, genetic testing, early intervention and routine follow-ups with specialized examinations are necessary for patients suspected with genetic syndromes. Nevertheless, medicine therapy such as growth hormone is still a challenge for those patients with short stature.

## Conclusion

In the present report, a *de novo* heterozygous variant within exon 16 of *KAT6B* (Chr10p: 76781966-76781967) was detected in a 7-months-old Chinese female infant with symptoms of short stature, global developmental delay, and clinical features consistent with SBBYSS. The correlation analysis of genotype-phenotype expands our understanding of the clinical genetics for *KAT6B*-associated disorders.

## Ethics Statement

Written informed consent was obtained from the patient's parents for the publication of this case report and any potentially identifiable images.

## Author Contributions

JS substantially contributed to the conception of this manuscript and critically revised the manuscript for important intellectual content. JS, LZ, and LL contributed to the acquisition, interpretation of the clinical data. DW was responsible for the testing, analysis and interpretation of the genetic data. LZ drafted the manuscript. All authors approved the final version.

## Conflict of Interest

The authors declare that the research was conducted in the absence of any commercial or financial relationships that could be construed as a potential conflict of interest.
